# The Utility of the Proximal Epiphysis of the Fifth Metatarsal in Age Estimation

**DOI:** 10.1111/1556-4029.12069

**Published:** 2013-02-20

**Authors:** Catriona M Davies, Lucina Hackman, Sue Black

**Affiliations:** ^1^ Centre for Anatomy and Human Identification, University of DundeeDundee, DD1 5EH, UK

**Keywords:** forensic science, forensic anthropology, age estimation, epiphyseal union, foot, fifth metatarsal

## Abstract

Radiographs of 277 living individuals were assessed via a numerical scoring system to determine the timing of appearance and degree of fusion between the proximal epiphysis of the fifth metatarsal and its diaphysis. The epiphysis was observed to first appear in females at 8 years and 10 years in males and fuse by 14 years in females and 15 years in males. When assessing the level of agreement of category assignment, inter-observer agreement was 78% for females and 64% for males whereas intra-observer agreement was 77% for females and 86.1% for males. These results suggest that the maturation of the proximal epiphysis of the fifth metatarsal may be of value in age estimation in the child and that the scoring system is sufficiently robust to merit continued investigation. Previously this epiphysis has been considered an inconstant feature, but this research confirmed its presence in all individuals studied.

In cases where incomplete human remains are recovered, it is crucial that all available information is used in an attempt to ascertain the identity of the individual (1). The foot and ankle are frequently recovered in isolation as a result of animal scavenging in terrestrial environments; or natural decomposition and disarticulation caused by the movement of water in fluvial or marine environments (2–5). The retention of the multiple skeletal elements of the lower limb can often be attributed to the protective and containing nature of footwear, including socks and shoes.

For estimation of chronological age to be recognized as a legally admissible method of forensic assessment, it is important that existing methodologies be reviewed regularly, and if necessary, alterations made to maximize all available information and increase scientific robusticity (6). Unlike methods of age assessment developed on other regions of the skeleton, for example the hand and wrist (7–10), estimation of chronological age from the foot and ankle has not been the focus of recent research to assess the accuracy or validity of available techniques. Following recommendations made by the Law Commission in 2011 (6) all methods of identification currently used may require re-evaluation if they are to be applied to cases under forensic investigation. Included in these recommendations is the obligation for all methods of forensic assessment to be based on statistically valid and robust methodologies to fulfill the requirements of evidentiary admissibility (6).

Age estimation in the foot and ankle, as in other areas of the body depends upon the appearance and fusion of epiphyses. The center of ossification, historically known as the Os Vesalium, has been recorded in the literature as a rare accessory ossicle of the foot similar in development to other ossicles including the Os Trigonum and Os Intermetatarsum (11),(12). Consequently, it has been dismissed by some as a relatively inconsistent center of ossification, cited as appearing in only 28% of the population (13). The frequency of appearance and the etiology of the ossification center however is disputed, as some studies have reported the presence of the epiphysis in all individuals examined (12–14). It is the view of some authors that the center of ossification located on the lateral aspect of the base of the fifth metatarsal is a traction epiphysis (15) sited at the attachment for the tendon of the *Peroneus Brevis* muscle (12),(13),(15).

There is a paucity of research regarding the utility of the proximal epiphysis of the fifth metatarsal for age estimation. Reference studies which quote the age ranges for the appearance of the epiphysis as *c*. 9–10 years in females and 12 years in males, with fusion occurring over the subsequent 2 years (16),(17); however these values are based on relatively few studies. As the original study examined a population in the 1930s (17), it is necessary to re-evaluate the timing of appearance and fusion of the epiphysis of the fifth metatarsal on modern material if it is to be used for forensic evaluation.

The objectives of this study therefore were to assess the prevalence rate of this center of ossification, and to ascertain the time of its appearance and subsequent fusion in male and female individuals from a modern clinical Scottish sample. This will clarify the utility of this center of ossification within the context of forensic age assessment of the foot.

## Materials and Method

A sample of radiographs representing 277 individuals consisting of 125 females and 152 males was obtained from Ninewells Hospital, Dundee. All females in the sample ranged between 6 and 14 years of age and all males ranged between 8 and 15 years of age. The sample images were collated from clinical radiographs of children admitted to Accident and Emergency at Ninewells Hospital and represented a cross-section of the subadult patient population of the Tayside NHS trust. As the radiographic images used were taken for clinical purposes, ethical approval was obtained from the University of Dundee and Tayside NHS Trust for their use in this study.

**Fig. 1:**
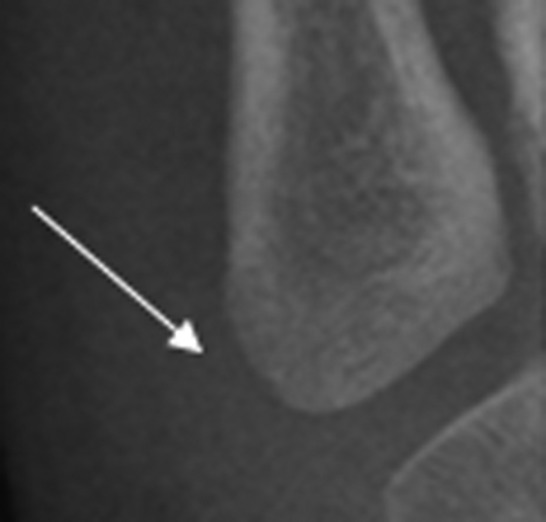
Example of maturity stage 0 in a female aged 7 years. Arrow highlights region where the future epiphysis will appear.

**Fig. 2:**
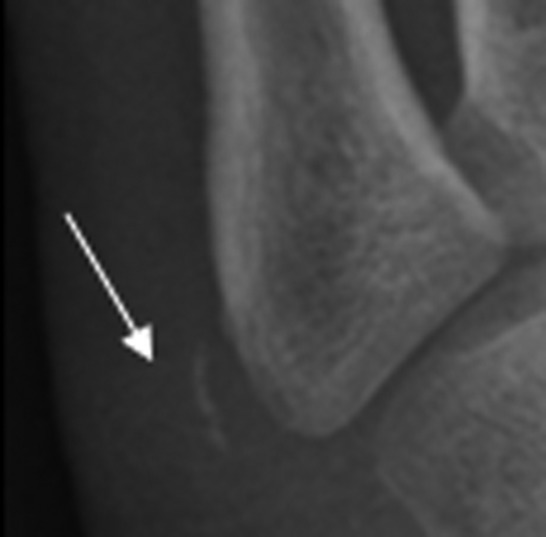
Example of maturity stage 1 in a male aged 11 years. Arrow highlights the ossified flake of the epiphysis.

**Fig. 3:**
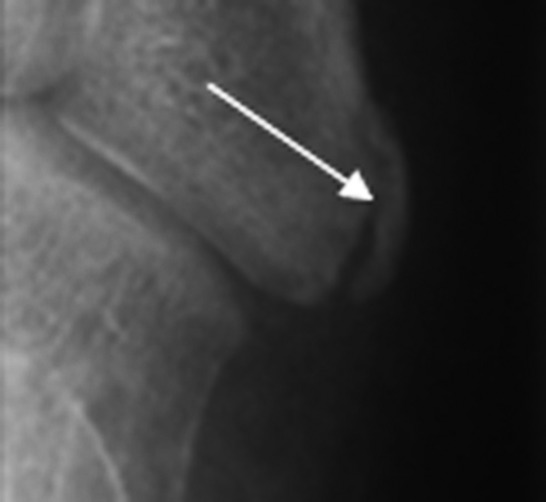
Example of maturity stage 2 in a male aged 13 years. Arrow highlights the epiphysis commencing fusion to the body of the metatarsal.

**Fig. 4:**
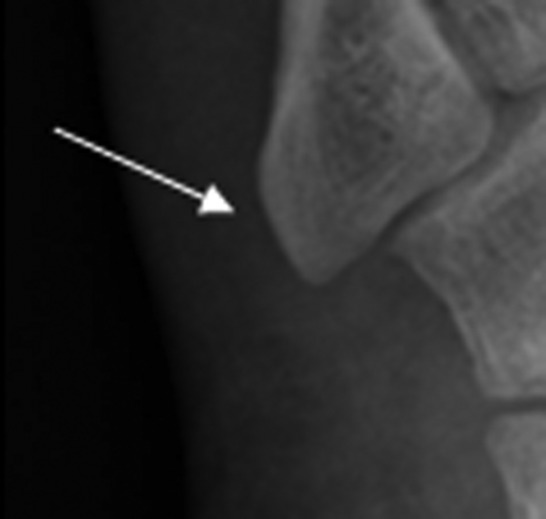
Example of maturity stage 3 in a male aged 17 years. Arrow highlights completed fusion of the epiphysis to the metaphysis.

To assess the stage of ossification and fusion of the proximal epiphysis of the fifth metatarsal quantitatively, a scoring method was developed, similar to those used by O’Connor et al. (18), Schmeling et al. (19), Schaefer and Black (20), and Whitaker et al. (21). Each individual radiograph was examined according to the criteria outlined below (summarized in Table 1). Figures 1–4 illustrate each of the four maturation stages.

**Table 1:** Scoring system for stage of ossification and fusion of the proximal epiphysis of the fifth metatarsal

0	Ossification center absent
1	Ossification center present, but fusion has not commenced
2	Fusion is ongoing
3	Fusion is complete and fusion line obliterated

### Criteria for Assigning Maturity Stages

#### Stage 0; Ossification Center is Absent

No radio-opaque center of ossification that would correspond to the proximal epiphysis is discernible in the region lateral to the base of the fifth metatarsal (Fig. 1). The outline of the lateral aspect of the body of the metatarsal is flattened and a small metaphyseal surface may be visible.

#### Stage 1: Ossification Center Present, but Fusion has not Commenced

At this stage, a small ossified flake can be seen lateral to the body of the fifth metatarsal (Fig. 2).

#### Stage 2: Fusion is Ongoing

The epiphyseal flake has started to fuse to the metaphysis of the metatarsal (Fig. 3). During this phase, small radiolucent spaces may be visible between the epiphysis and the metaphyseal surface.

#### Stage 3: Fusion is Complete and the Epiphyseal Line is Obliterated

The epiphysis has fully fused to the body of the metatarsal, the outline of which appears more rounded and the projection at the base of the bone is larger than observed in Stage 2 (Fig. 4). No epiphyseal scar remains.

Each radiograph was assessed by the first author, and the maturity stages were recorded. The data were subsequently grouped by sex and known chronological age. The percentage of each stage found at each chronological age and the prevalence of the epiphysis within each age cohort for females and males was calculated and tabulated (Tables 2 and 3).

**Table 2.** Percentage of female individuals exhibiting each stage of ossification

	% of Female Individuals in Each Age Group Displaying Each Stage of Ossification (*n* = 125)				
	Stage				
Age (Years)	0	1	2	3	Total % of Cohort Exhibiting an Epiphysis
6 (*n* = 14)	100	0	0	0	0
7 (*n* = 3)	100	0	0	0	0
8 (*n* = 11)	81.8	0	18.2	0	18.2
9 (*n* = 16)	62.5	37.5	0	0	37.5
10 (*n* = 18)	44.5	11.1	33.3	11.1	55.5
11 (*n* = 19)	0	36.8	31.6	31.6	100
12 (*n* = 18)	0	16.7	22.2	61.1	100
13 (*n* = 14)	0	7.1	0	92.9	100
14 (*n* = 12)	0	0	0	100	100

**Table 3.** Percentage of total male individuals exhibiting each stage of ossification

	% of Male Individuals in Each Age Group Displaying Each Stage of Ossification (*n* = 152)				
	Stage				
Age (Years)	0	1	2	3	Total % of Cohort Exhibiting an Epiphysis
8 (*n* = 6)	100	0	0	0	0
9 (*n* = 6)	100	0	0	0	0
10 (*n* = 13)	76.92	23.08	0	0	23.08
11 (*n* = 25)	52	28	12	8	48
12 (*n* = 40)	20	27.5	45	7.5	80
13 (*n* = 27)	18.52	14.81	40.74	25.93	81.48
14 (*n* = 19)	5.26	5.26	26.32	63.15	94.74
15 (*n* = 16)	0	0	0	100	100

The suggested age range during which the epiphyseal flake at the proximal tuberosity of the fifth metatarsal appears and fuses was derived from the results of preliminary assessments carried out by the first author. Following the primary analyses, female individuals of 6 and 7 years of age, and male individuals of 8 and 9 years of age were removed from the inter-observer sub-sample due to the absence of ossification in the region of the proximal epiphysis of the fifth metatarsal in all individuals examined.

Inter-observer error was assessed using a subset of radiographs comprising 25 male and 23 female randomly selected individuals that were assessed by the second author using the criteria outlined in Table 1.

Intra-observer error was assessed using the radiographs of six individuals from each year cohort which were randomly selected for re-assessment by the first author 2 months after the first round of assessment. This resulted in 36 males and 42 females in the intra-observer test sample. These were re-assessed using the criteria outlined in Table 1.

### Statistics

Statistical analyses of the data were performed using Sigmaplot 12.0™ (Systat Software Inc., Chicago, IL) and Microsoft Excel 2010™(Redmond, WA).

## Results

Tables 2 and 3 illustrate the percentage of each stage of ossification and fusion found within individual age cohorts and the prevalence of the epiphysis within age cohorts for females and males, respectively. The distribution of individuals shown in these tables suggests that there is a progressive pattern to the ossification and fusion of this center in both males and females that is related to the chronological age of the individual.

### Females

Table 2 illustrates the age ranges associated with each stage of maturity according to the results of this study. The youngest subjects in whom the epiphysis was observed were 8 years of age. Active fusion of the epiphysis to the metaphysis was observed in subjects between 8 and 12 years of age. Complete fusion of the epiphysis was first observed in subjects of 10 years of age and was completed in all subjects by 14 years of age.

The prevalence of the epiphysis within each female age cohort is also presented in Table Table 2. These results show that while the epiphysis does not appear in all individuals at exactly the same time, the development of the epiphysis proceeds at a relatively predicable rate. This is evidenced by the variability in the presence of an ossified flake between 8 and 10 years of age, compared with the consistent observation of an epiphysis in all individuals within each cohort from 11 years of age to 14 years of age.

### Males

Table 3 illustrates the age ranges associated with each stage of maturity according to the results of this study. The epiphysis was first observed in male subjects at 10 years of age and was present as a separate center between the ages of 10 and 14 years. Active fusion of the epiphysis to the metaphysis was first observed in subjects aged 11 years, but occurred in males between 11 and 14 years. Complete fusion of the epiphysis was first observed in subjects of 11 years of age and fusion was complete in all male subjects by 15 years.

The prevalence rates of the proximal epiphysis of the fifth metatarsal for males within each age cohort are also presented in Table 3. These results show a steady increase in the prevalence of the epiphysis within progressive age cohorts. In contrast with female individuals, the only age cohort in whom a prevalence rate of 100% was found was the 15 years of age cohort.

Table 4 shows the age ranges for the appearance and fusion of the epiphysis in both males and females. If the sex of the subject is not known, then the age ranges must be combined, inevitably leading to a much wider potential age range.

**Table 4.** Age ranges against maturity stages for males, females, and remains of unknown sex

	Maturity Stage			
Sex	0	1	2	3
Female	≤10	9–13	8–12	≥10
Male	≤14	10–14	11–14	≥11

Absence of the epiphysis would be indicative of female individuals who are less than or equal to 10 years of age, or male individuals who are 14 years of age or younger. With remains of unknown sex, the absence of an epiphysis would indicate an individual who is younger than or equal to 14 years of age. If the epiphysis is present, but not actively fusing, it is indicative of a female between 9 and 13 years and a male between 10 and 14 years of age. If the sex of the remains is not known then the individual may be between 9 and 14 years.

If the epiphysis is actively fusing, this would indicate a female of between 8 and 12 years, and a male of between 11 and 14 years. If sex is not known, an individual with a fusing epiphysis will likely be between 8 and 14 years of age.

If the epiphysis is fully fused then a female is likely to be equal to, or older than, 10 years and a male will be equal to, or older than, 11 years. If the sex of the remains is not known, fusion of the epiphysis will represent an individual who is at least 10 years of age.

### Inter-Observer Analysis

**Fig. 5:**
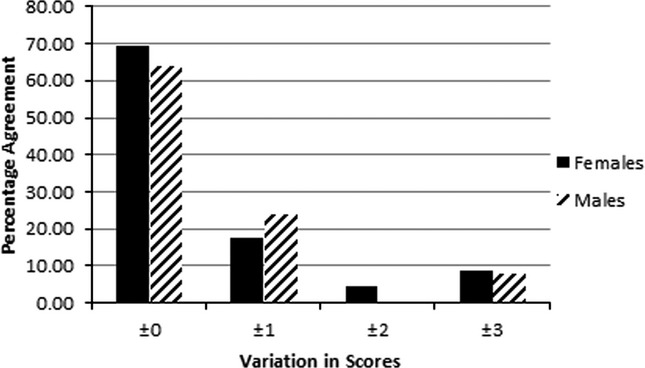
Difference between the observer scores for inter-observer analysis as a percentage of the total inter-observer assessments.

Analysis of the data obtained from the assessments carried out by the first and second authors was undertaken to test the consistency of the method. The percent agreements between the observations of the authors for the male and female samples are presented in Fig. 5. These assessments suggest that multiple observers are likely to assign the same score to an individual on 78.3% of occasions for females, and 64% of occasions for males.

Of the seven females for whom the scores attributed by the observers differed, 57.1% of disagreements were by one stage, 14.3% differed by two stages, and 28.6% of discordant results differed by three stages. Of the nine males for whom assessments differed, 66.6% did so within one stage. The remainder of the discordant results diverged by three stages. Upon further analysis of these results, a pattern of overestimation emerged in the distribution of the discordant data. Within both the female and male results, the scores attributed by the second author were more likely to be in excess of those assigned by the first author, with the exception of those occasions when the scores differed by three stages.

### Intra-observer Analysis

**Fig. 6:**
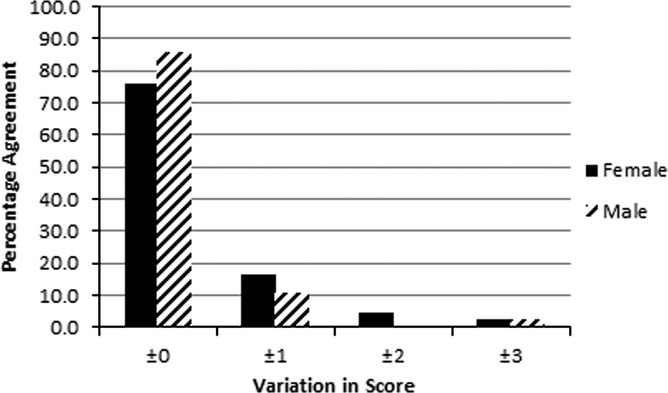
Difference between the scores assigned during intra-observer analysis as a percentage of the total intra-observer assessments.

Analysis of the intra-observer data was undertaken to determine the degree of agreement between the first and second assessments carried out by the first author. The percent agreements between the first and second rounds of assessment carried out on the male and female samples are presented in Fig. 6. These assessments suggest that repeated observations carried out by the same individual will yield the same score on 77.2% of occasions for females and 86.1% of occasions for males.

Of the 10 cases where the scores assigned to female individuals were discordant, 70% of disagreements differed by a single stage, 20% differed by two stages, and 10% differed by three stages. Within the male sample, of the five cases where discordant results were obtained, 80% were within a single stage. In the remaining 20% of assessments, scores were found to vary by three stages. When these data were analyzed further, it became apparent that the scores assigned in the second round of observation were more conservative than those assigned during the preliminary observations as the first author was more likely to assign a lower score to the individuals within the sample. The only exception to this was found in the female sample where scores which differed by one were more likely to be assigned a higher score during the repeat assessment.

## Discussion

The lower limb is an anatomical region frequently recovered in cases of partial remains, particularly those involving fluvial or marine environments (2,4,5). This bias in discovery of elements could be due to the natural disarticulation sequence, during which limbs become detached from the torso (22), combined with the protection afforded by footwear which may be sufficient to retain all the skeletal elements *in situ*. The consequential reliance on assessments of identity based on only one body part supports the need for constant validation of methods of assessing skeletal age based on the region of the foot and ankle.

The appearance and fusion of the center of ossification historically known as the “Os Vesalium” or the “Bone of Vesalius” is not currently included as a maturity criterion in forensic age assessment, although its fusion times are recorded in the Radiographic Atlas of Skeletal Development of the Foot and Ankle (17). Now accepted as a traction epiphysis of the tuberosity located at the proximal end of the fifth metatarsal (11), the potential utility of the timing of appearance and fusion of this center of ossification within forensic age assessment has remained largely unquantified.

An investigation into the timing of appearance and the rate of fusion of the proximal epiphysis of the fifth metatarsal was undertaken using a numerical scoring method, similar to that used by Schaefer and Black in their study of Bosnian males 20 among others (19,23–26). As noted by Whitaker et al. (21), when applying this methodological approach it is imperative that the number of stages used allows the variation within a sample to be shown while minimizing the effect of observer interpretation. For the purposes of this study, four stages of ossification were thought appropriate. Fewer stages would equate to less precision when examining the ossification and fusion phases, and therefore could potentially increase inter-observer and intra-observer errors. A greater number of stages could introduce more ambiguity into the assessment by increasing the effect of observer interpretation of the descriptive criteria.

Through the application of these criteria, a well-defined pattern was observed in the timing of appearance of the proximal epiphysis of the fifth metatarsal in both male and female individuals in the population studied. The appearance and fusion of the epiphysis commenced later in males than in females, but showed activity over a similar duration. This supports the anthropological and medical literature where the precocity of female development is widely reported (16,17,27–30. The results of this study suggest that the appearance and fusion of the epiphysis for the proximal fifth metatarsal is consistent with other centers of ossification with regard to the precocious development of females by being *c*. 2 years in advance of males (31,32).

It is widely acknowledged that sex determination from juvenile remains is problematic (33). Due to the precocious development of females relative to males, and the difficulties posed by sex determination in children, it is necessary to present wider, non-sex-specific age ranges for use in cases where the sex of the remains is not known. In cases such as these, a composite age range which encompasses both male and female age ranges should be considered. Although the timing of appearance and fusion may show some considerable variation, the results can be summarized in their most basic form as follows:

- Absence of an epiphysis indicates a child aged 10 years or younger.
- Presence of an epiphysis at any stage (separate, fusing, or fused) indicates a child 10 years or older.

Due to the cross-sectional nature of this study, it is important to note that the absence of a visible epiphyseal flake in the female aged 14 and male aged 15 cohorts is interpreted as the completion of fusion. As the prevalence of the epiphysis is a matter of contention within the literature (12–14), it is necessary to note that the absence of an epiphysis in an otherwise skeletally mature foot could be due to an epiphysis never having formed. A longitudinal study would be required to clarify this.

In the case of a skeletally immature foot void of soft tissue, it is likely that the absence of an epiphysis would be due to problems in identification or recovery. In cases such as these, it may be theoretically possible to ascertain if an epiphysis had appeared through morphological analysis of maturational changes in the metaphyseal surface of the proximal end of the fifth metatarsal. As this study was solely concerned with radiographic observations, an additional study would be required to test this hypothesis.

The results derived from this study broadly support the timings of appearance suggested by the extant literature regarding the epiphysis of the proximal fifth metatarsal (16,17). Scheuer and Black (16) state that the epiphysis appears between 9 and 10 years in females, and 12 and 13 years in males, and fuses over the following 24 months. As this text was based on available published literature, these values represent the most commonly cited ages for this maturation stage. The Radiographic Atlas of Skeletal Development of the Foot and Ankle by Hoerr et al. (17) states that the epiphysis was found to appear at 9.7 ± 1.2 years and fuse at 11.7 ± 1 year in females; and appear at 12.1 ± 1.3 years and fuse at 14.2 ± 1.1 years in males within their research sample. These values are consistent with the results obtained from this study.

Intra-observer agreement was found to be higher than interobserver agreement in the analysis of male individuals, whereas for females the percent inter-observer agreement was found to be 1.1% higher than the intra-observer agreement rate. It would be reasonable to expect intra-observer agreement to be higher than inter-observer agreement due to the influence of experience (34). As the first author completed the assessments of the full data set prior to completing the intra-observer assessments a greater degree of experience in both application of the method and interpretation of the radiographs was achieved.

In the instances where the scores assigned to an individual differed either between observers or between observations, the majority of scores fell within one stage of that provided on the first occasion by the first author. In the context of the age ranges associated with each score, variation by one stage is acceptable as it does not substantially alter the projected age range for the individual (see Table 4).

Within both the inter-observer and intra-observer analyses, two stage variations were observed within the female samples. Of the three instances in which this was found, two of the disagreements related to the difference between stage 1 and stage 3. This could be explained by the quality of the image on which the assessment was being made and the interpretation of the epiphysis as a radiographic artifact. It could also be explained by a failure to note the presence of the epiphysis and consequently mistaking the metatarsal as being fully mature. On the single occasion when a two stage variation was recorded between a stage 0 and a stage 2 the variation could be attributed to human error. As this is a subjective method of assessment, human error cannot be eradicated completely, it can only be minimized.

Occasionally, a three stage variation was found between observers or observations, relating to the difference between stage 0 and stage 3. This can be explained by the misclassification of an epiphysis, which has fully fused as one which is yet to form. This is understandable as the gross morphology of the immature base of the fifth metatarsal as observed prior to the appearance of the epiphysis can be similar to a mature bone to which fusion of the epiphysis has completed.

It would be expected that with greater experience in the radiographic assessment of immature fifth metatarsals, the proportion of disagreements within the inter-observer and intra-observer assessments would decrease, resulting in increased accuracy (34). This would also bring the method into line with the recommendations included in the Law Commission report which require evidence proffered for admission to fulfll the requirement of evidentiary reliability through stability of application (6).

The absence of a significant difference between the results obtained from the male and female analyses suggests that the criteria are equally applicable to both sexes in the study sample, in contrast to some methods of forensic assessment based on skeletal collections which exhibit a sex bias in the accuracy of the results due to sex biases present in the reference population (35). Further testing on alternative sample populations is required to further validate the findings of this initial study.

The radiographic nature of the images used in the assessment of appearance and fusion of the proximal epiphysis of the fifth metatarsal necessitate that the proposed age ranges are only applied to radiographic material. Due to the small size of the epiphysis, it is likely that it would be lost or not recovered in cases involving dry bone. This is supported by the literature as the epiphysis can be seen in the radiographic study carried out by Hoerr et al. (17), however, it was considered an extremely rare epiphysis in previous studies which examined dry bone (13), it was therefore not included as a maturity indicator in these publications.

As this is a visual method of age estimation, the interpretation of the radiograph and the subsequent application of the maturity criteria by an observer introduce a level of subjectivity to the assessment. To reduce this effect, the number of maturity stages was minimized to reflect only the appearance of the ossification center, and the duration and timing of complete fusion. Through the application of precise descriptions, the maturity criteria are open to minimal interpretation, resulting in consistent application of the method by different observers.

As the data used in this study are radiographic and cross-sectional, it represents only a snapshot of the patient population of NHS Tayside. The study sample was not screened for ethnicity or other discriminating factors. The population on which the research was carried out can therefore be considered to reflect the general patient population of Tayside.

The effect of ancestry on the rate of skeletal development has been discussed in the literature (36,37), however, it is generally accepted that the differences observed are related less to genetic differences, and more to environmental factors such as socio-economic status (37–39). Consequently, it is necessary to take these factors into account when interpreting the results. Dundee City local authority exhibits the highest percentage of the population living in relative poverty (24%) in Scotland, as defined by the Scottish Government in 2010 (40), compared with 19.4% in Angus, and 16% in Perth and Kinross (40). The cross-sectional nature of this study necessitates the assumption that an individual from one socioeconomic background is as likely to require treatment at Accident and Emergency as any other. The overrepresentation of individuals of low socioeconomic status or poor health would likely bias the results. To quantify the impact of socioeconomic status on the fusion of the fifth metatarsal, it would be necessary to complete a research study using groups of known socioeconomic status.

## Conclusion

Through the visual assessment of radiographic images of the region surrounding the proximal tuberosity of the fifth metatarsal, the timing of ossification and fusion of the proximal epiphysis of the fifth metatarsal has been shown to be consistent within sex groups.

In female subjects, the epiphysis was discernible on radiographs from 8 years of age. The ossification of the epiphyseal center continued in individuals until 13 years of age and fusion to the diaphysis was evident between the ages of 8 and 12 years. Complete fusion of the epiphysis to the metaphyseal surface of the diaphysis was first observed in subjects aged 10 years and was complete in all females by 14 years of age.

In male subjects, the epiphysis was not observed before 10 years of age. The center appeared between 10 and 14 years of age, with active fusion between 11 and 14 years. Complete fusion of the epiphysis to the metaphyseal surface of the diaphysis was first observed in subjects of 11 years of age, up to 15 years of age, at which point fusion was complete in all male subjects. In addition to sex-specific age ranges, estimations for each maturity stage for an individual of unknown sex are presented. Although the epiphysis may show considerable variation in its appearance and fusion, the results of this study can be summarized as the absence of an epiphysis indicates an individual of 10 years of age or younger; the presence of an epiphysis at any stage of maturation indicates an individual of 10 years or older.

The scoring system developed for use in the assessment of the ossification and fusion of the proximal epiphysis of the fifth metatarsal has proved to be of sufficient repeatability and reliability within a modern Scottish population to warrant further investigation. It is therefore in compliance with the recommendations set out by the 2011 Law Commission Report (6).

The approach presented in this article is not intended as the sole method for age estimation from the foot, nor is it intended to replace any currently applied methodologies. This method is intended for use as an additional source of information, which may aid the forensic practitioner in estimating the chronological age of an individual, when presented with the distal lower limb segment in isolation. When concerned with fragmented or partial remains, it is imperative that methods exist to allow the maximum amount of information to be recovered in the pursuit of a biological profile. It is hoped that this method will aid in the pursuit of this goal.
